# Limited Dispersal and Significant Fine - Scale Genetic Structure in a Tropical Montane Parrot Species

**DOI:** 10.1371/journal.pone.0169165

**Published:** 2016-12-29

**Authors:** Nadine Klauke, H. Martin Schaefer, Michael Bauer, Gernot Segelbacher

**Affiliations:** 1 Animal Ecology and Evolution, Faculty of Biology, University of Freiburg, Freiburg, Germany; 2 Wildlife Ecology and Management, Faculty of Environment and Natural Resources, University of Freiburg, Freiburg, Germany; Deakin University, Australia, AUSTRALIA

## Abstract

Tropical montane ecosystems are biodiversity hotspots harbouring many endemics that are confined to specific habitat types within narrow altitudinal ranges. While deforestation put these ecosystems under threat, we still lack knowledge about how heterogeneous environments like the montane tropics promote population connectivity and persistence. We investigated the fine-scale genetic structure of the two largest subpopulations of the endangered El Oro parakeet (*Pyrrhura orcesi*) endemic to the Ecuadorian Andes. Specifically, we assessed the genetic divergence between three sites separated by small geographic distances but characterized by a heterogeneous habitat structure. Although geographical distances between sites are small (3–17 km), we found genetic differentiation between all sites. Even though dispersal capacity is generally high in parrots, our findings indicate that dispersal is limited even on this small geographic scale. Individual genotype assignment revealed similar genetic divergence across a valley (~ 3 km distance) compared to a continuous mountain range (~ 13 km distance). Our findings suggest that geographic barriers promote genetic divergence even on small spatial scales in this endangered endemic species. These results may have important implications for many other threatened and endemic species, particularly given the upslope shift of species predicted from climate change.

## Introduction

Habitat loss and fragmentation have been identified as the major threats to species extinction [1]. Habitat loss and fragmentation can cause population decline through the overall reduction of total suitable habitat or by disrupting dispersal pathways leading to the loss of genetic connectivity between fragments and to genetic depletion in small, isolated populations [2–5]. Yet, the negative effects of habitat loss and fragmentation on population persistence will depend on the amount and size of suitable habitat remnants, and on the ecological and life-history traits of a given species [6,7]. As data on key ecological traits such as dispersal behaviour and habitat specialization as well as the genetic structure of populations are often lacking, particularly for species inhabiting the tropics, it has been challenging to predict population viability in tropical biodiversity hotspots in the face of rapidly proceeding environmental change [8,9].

Mobile organisms like birds are usually expected to disperse over large distances and show corresponding low levels of genetic differentiation between neighbouring populations [10–12]. In contrast to temperate bird species, there is increasing evidence that many tropical bird species show low dispersal propensities [13,14]. However, most studies on dispersal behaviour in tropical birds are based on observational data and capture-recapture methods [8,14,15] and therefore do not directly assess the level of effective gene flow between populations. Moreover, low dispersal propensities have been associated with high levels of ecological specialization in tropical species [16]. Most information on dispersal and gene flow is available for habitat specialists showing *per se* low mobility, like understorey birds [17–23], whereas knowledge about fine-scale genetic structure of highly mobile habitat specialists is as yet scarce.

In addition to ecological specialization, landscape characteristics, like mountains, valleys or water bodies influence dispersal decisions of species and thus are reflected in their genetic population structure [24–27]. Allopatric speciation events through the development of geographic barriers such as valleys, play an important role in explaining the high species diversity in the Andes [28–31]. In those montane forests many species occupy special habitats which are confined to narrow altitudinal ranges and often have very limited spatial extensions. In birds, Weir [32] found high genetic differentiation between several Andean bird populations separated by relatively broad lowland barriers like river valleys. Importantly, human-induced fragmentation can enhance such barriers, particularly in narrow altitudinal ranges. As a consequence, populations may differentiate even on small spatial scales if species do not cross unsuitable habitat. Knowledge about effective dispersal rates and the fine-scale genetic structure of populations across natural barriers in heterogeneous environments such as the Andes will thus provide valuable insights into how these factors affect population connectivity and persistence in the biodiversity hotspots of tropical mountain ranges.

In this study, we investigate the fine-scale genetic structure in the endangered El Oro parakeet (*Pyrrhura orcesi*). The El Oro parakeet is endemic to southwest Ecuador and is considered to have undergone a severe bottleneck during the last decades [33]. Several Pyrrhura species are globally threatened as they are confined to small distributional ranges. Pyrrhura parakeets are considered to be highly sensitive to landscape structure and to depend on closed canopy cover for long-distance dispersal [34]. The El Oro parakeet is a frugivorous species inhabiting cloud forest [35] occurring in a narrow elevational range (800 m– 1300 m) at the western foothills of the Andes in southwest Ecuador [33]. Accordingly, its distributional range is extremely small, corresponding to an area of approximately 750 km^2^. Because of that small range, it is important to understand whether natural and human-induced landscape characteristics influence population genetic structure in El Oro parakeets on a narrow geographic scale.

**Fig 1:**
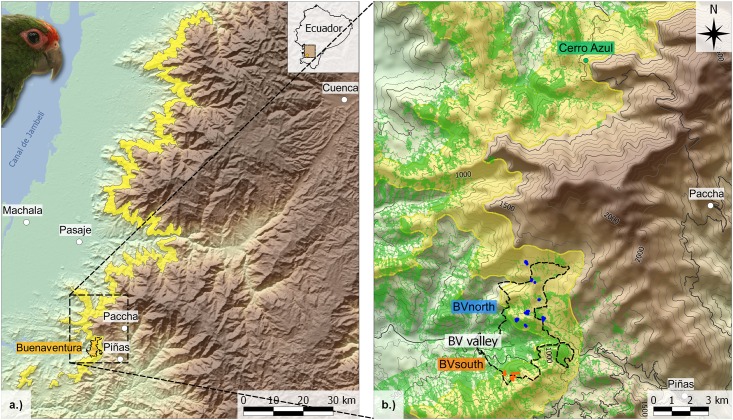
Map of the global distribution range of El Oro parakeets and the study sites. (a) The global distribution range specified by the yellow area is based on recent population monitoring data of Cesar Garzon (marked elevational range: 800–1400 m). The dashed square specifies the study area. In the right-hand corner, the spatial location of the study area within Ecuador is shown. (b) Sampling locations at the southern (orange circles) and northern part (blue circles) of Buenaventura reserve and Cerro Azul (green circle). Dashed line in (b) specifies the borders of Buenaventura reserve. The Buenaventura valley separates BV_South_ and BV_North_ and lies outside the distribution range of parakeets (yellow area). Forested areas are highlighted in light green and inferred from geo-referenced satellite images (RapidEye, Blackbridge, Germany) taken in 2010 and 2013. Contour lines are given in 500 m steps.

Fine-scale genetic structure was investigated for the two largest known subpopulations of the El Oro parakeet, located within the Buenaventura reserve and near Cerro Azul (Fig 1). Genetic and observational data were sampled from three sites separated by three to 17 km distance, which allowed us to compare gene flow and dispersal events on varying spatial scales. The study area is characterized by a variable habitat structure with the Buenaventura reserve being divided by a largely forested valley ranging down to 450 m. In contrast, neighbouring areas are higher than 1000 m but are largely deforested (Fig 1). This variability in habitat structure between our study sites allowed us to infer the importance of landscape characteristics for genetic connectivity of El Oro parakeet populations. We assessed recent dispersal rates between and within our study populations based on the genetic data of 249 individuals and on observational data from four years. Genetic divergence between the three study sites was evaluated on the population-level as well as based on individual genotypes. We further tested whether genetic divergence between the study sites might be explained by geographic distance. Finally, we estimated the extent to which the El Oro parakeet subpopulations are affected by habitat loss applying two different bottleneck analyses.

## Materials and Methods

### Ethics statement

Field work procedures and data sampling were reviewed and approved by the Ministerio del Ambiente, Ecuador (permission No. 012–09 IC-FAU-DNB/MA; 002-IC-FAN-DPO/MAE). This permission allowed for the collection of blood samples. No animals were killed for this study and handling time was kept to a minimum. Field work was carried out in the protected area of the reserve Buenaventura and a privately owned area at Cerro Azul. Permission to conduct field work in these areas was granted by the Fundación Jocotoco and the land-owner of the area at Cerro Azul, respectively. Sample collection was reviewed and approved by the Ministerio de Agricultura, Ganadería, Acuacultura y Pesca, Ecuador (permission No. 0060553, 0001175 and 04836).

### Study species and distribution range

The El Oro parakeet is a facultative cooperative breeder usually nesting in extended family groups with one central breeding pair [36]. The distributional range of El Oro parakeets stretches from the canton of Marcabelí in the province of El Oro near the Peruvian border in the south to the northernmost population of Molleturo in the canton of Cuenca in the province of Azuay (Fig 1a). It was first described in 1988 at the locality of Buenaventura [37]. There, it was originally found to inhabit elevations of 600–1100 m with a single record at 300 m in 1939 [37]. Ten years ago, the species was regularly found in Buenaventura only above 800 m and up to 1300 m [35]. Within the last three years, parakeet groups have continued to move to higher elevations being increasingly observed up to 1600 m (pers. obs.).

Ecuador exhibits the largest deforestation rate of South America with no more than 4% of the original forest cover remaining at the western coastal areas and the pre-montane regions [38]. This immense habitat loss is expected to have led to a strong population decline of the El Oro parakeet population during the past decades. Recent population size estimates are 250–1000 mature individuals [33]. Yet, the effective population size might be considerably lower given the complex social breeding system with many adults not reproducing in a given year. Although El Oro parakeets are highly dependent on forest habitat for foraging and old tree stock for breeding, they are known to tolerate some habitat fragmentation and can be frequently observed crossing narrow (< 500 m) deforested areas ([32], pers. obs.). However, the effects of habitat loss and fragmentation on long-distance dispersal and population connectivity are not yet known.

### Study populations

Sampling took place at the Buenaventura reserve (2200 ha) and at Cerro Azul (~1600 ha), which is located approximately 17 km northeast of Buenaventura (Fig 1b). In 2006, a nest box scheme has been started at the Buenaventura reserve to provide nesting opportunities to the parakeets. Since then parakeets have continually occupied nest boxes. Our two study populations are the so far largest known subpopulations of the El Oro parakeet harbouring approximately 240 individuals in Buenaventura and 80–100 individuals in Cerro Azul (*censu* Cesar Garzon). The regions of Buenaventura (BV) and Cerro Azul (CA) are connected by a continuous mountain range that lies within the typical elevational range of El Oro parakeets (Fig 1b). The area between these regions is characterized by humid cloud forest patches interspersed by pastures used for cattle ranging or by mining areas. In contrast, the northern (BV_North_) and southern (BV_South_) areas of Buenaventura are divided by a valley with an elevation of 450–500 m, which is clearly lower than the current parakeet’s elevational range. Note, however, that El Oro parakeets were observed in the valley at just slightly higher elevations (~ 600 m) when they were discovered 30 years ago. Throughout the valley, a narrow, continuous forest patch connects the northern and southern slopes of BV. The valley provides the shortest dispersal pathway between BV_North_ and BV_South_ (approximately 3 km) and parakeets could potentially cross it in a continuous forest layer (Fig 1b). In contrast, only small islands of forested areas remained at an elevation between 800 m and 1300 m near the eastern borders of the reserve and extensive cattle ranging is ongoing beyond them.

### Sampling scheme

Although observational data indicate that a few parakeets (~ 25 individuals) also reside in the area between CA and BV, poor infrastructure and rough terrain impeded capturing them. All samples were taken from the large populations at CA and BV. Parakeets were captured with mist nets or within nest boxes equipped with trap doors. Because of intensive population monitoring over a study period of four years (2009–2012), the majority of individuals of the Buenaventura population have been sampled. We banded and took blood samples of 233 individuals in Buenaventura (n_BVNorth_ = 126; n_BVSouth_ = 107). Of these individuals, 52 (n_BVNorth_ = 24; n_BVSouth_ = 28) were breeders in the study population. In CA, 16 individuals were banded and blood sampled in the years 2010 and 2011. GPS points were taken from every location where parakeets were captured. All parakeets were captured at an elevation between 800–1300 m (Fig 1b). Sample sizes between our two focal populations differ because of a behavioural study simultaneously conducted in Buenaventura [36,39].

High relatedness between individuals of a breeding group may bias our results. To avoid overestimating relatedness due to our sampling scheme and to meet prerequisites of random sampling, a reduced data set consisting solely of the 52 breeders captured in BV and 13 adults (excluding three juveniles) captured in CA was used in addition to the complete data set of n = 249 for analysing genetic divergence on the population- and the individual-level. As sample sizes among our study sites were similar for the reduced data set, this procedure also ensured that our results were not biased by sample size effects. Because results were consistent irrespective of which data set was used, most results inferred from the reduced data set are given in the supporting information section. If not stated otherwise, individuals that were captured several times were assigned to the location where they were captured last. This procedure ensured that all dispersal events observed between our study sites entered the analyses.

### Microsatellite genotyping

Blood samples were stored in 96%-Ethanol at -22°C. DNA was extracted with the DNeasy Blood and Tissue Kit (Qiagen, Hilden, Germany) following customers instructions. We used a set of 18 microsatellite markers for genotyping of individuals. Genotyping was performed with an ABI Sequencer 3130xl (Applied Biosystems, HITACHI, Foster City, USA) using the software GeneMapper ID-X 1.1 (Applied Biosystems). For description of the polymerase chain reaction (PCR) procedure as well as detailed information about marker characteristics see [36]. We did neither detect consistent deviations from linkage equilibrium between our markers when tested with several subsets of the data set nor did markers deviate from Hardy-Weinberg equilibrium [36].

### Genetic diversity

To estimate the genetic diversity maintained within the northern and southern parts of Buenaventura as well as within Cerro Azul, we calculated the observed heterozygosity (H_o_), the expected heterozygosity (H_e_) and the fixation index (F_IS_) with the software GenALEx 6.5 [40]. To account for differences in sample sizes between our sample sites, we calculated allelic richness (AR) with the software FSTAT 2.9.3 [41]. The analyses were based on the complete data set of n = 249 individuals.

### Population genetic structure

#### Genetic differentiation between local populations based on G_ST_ and D

To test whether genetic exchange between our study localities is sufficiently high to prevent differentiation between them, we estimated genetic differentiation as Jost´s D [42] and G_ST_. We calculated both indices of genetic differentiation to exclude that the observed differentiation is due to the statistical nature of one of these indices [42,43]. The bias-corrected estimators were used for both indices (D_est_ [42]; G_ST_est_ [44]). Genetic differentiation was calculated with the R package *Diversity* [45] which calculates the harmonic mean of differentiation indices across all loci. A randomization test with 1000 bootstraps was conducted to estimate the 95% confidence intervals.

#### Genetic clustering analyses

We tested the extent of genetic sub-structuring within our study population by applying a Bayesian clustering algorithm implemented in the software STRUCTURE ver. 2.3.4 [46]. This individual-based clustering method estimates the most probable number of genetic clusters K and, in contrast to the indices of genetic differentiation, makes no *a priori* assumptions about the population structure. It thus gives an unbiased estimation of potential genetic sub-structuring within the study sample [47]. The simulation was run with the CAR admixture model assuming correlated allele frequencies. We set the number of expected clusters K from 1 to 20 for the complete data set of 249 individuals and K from 1 to 8 for the reduced data set (n = 65). The maximum number of clusters was set to 20 for the complete data set because preliminary runs revealed no convergence for K = 8. We ran 15 iterations for each K with 10^6^ MCMC (Markov Chain Monte Carlo) steps discarding the first 2 ∙ 10^5^ steps. Simulations were run with and without prior information on sampling location. If sample location was used as prior, individuals were assigned to their hatching place or, if hatching place was unknown, to the place where they were captured first. The most likely number of clusters was inferred by the highest mean posterior likelihood and the highest delta K [47] calculated with Structure Harvester [48]. The average probability of membership Q to a specific cluster was calculated for each individual over 15 iterations with the software CLUMPP ver. 1.1.2 [49] and illustrated with the software DISTRUCT ver. 1.1 [50].

Additionally to the Structure analyses, we applied a spatially explicit model implemented in the software GENELAND ver. 4.0.5 [51], which incorporates individual genotype data together with spatial information of each individual. To infer the most probable number of clusters K in our study populations, we first made ten independent runs allowing the number of clusters K to vary. We let K vary from one to six for the reduced data set (n = 65) and from 1 to 20 for the complete data set (n = 249). We supposed a correlated allele frequency and set the number of MCMC to 2 ∙ 10^5^ iterations with a thinning interval of 100 iterations and a burn-in period of 200 iterations. The number of K was then inferred from the modal number of K given by these ten runs. In a second step, we made 30 runs keeping K fixed to the modal number of K to assign each individual to one of the genetic clusters. For this second step, we lowered the number of MCMC iterations to 1 ∙ 10^5^ with a thinning interval of 100 iterations and a burn-in period of 200 iterations. From these 30 runs, we chose the five best runs according to their mean log posterior probability. The average probability of membership to a specific cluster was then calculated based on these five runs for each individual over 1000 iterations with the software CLUMPP ver. 1.1.2 [43] and illustrated with the software DISTRUCT ver. 1.1 [44].

#### Isolation by distance

To further test whether genetic differentiation between the study sites can be explained by geographic distance, we tested for a correlation between geographic and genetic distance separately for individuals of Buenaventura and for individuals of Buenaventura and Cerro Azul. We measured the extent of isolation by distance (IBD) by conducting a Mantel test using pairwise linear genetic distance and geographic distance as implemented in GenALEx ver. 6.5 [38]. Geographic distance between individuals of BV was measured as straight line across the valley. In contrast, geographic distance between individuals of BV and CA was measured along an elevational belt lying within the parakeets’ elevational range at the western slope of the mountain between BV and CA (see Fig 1b). Geographic distance was thereby measured by applying the pathway function in googleEarth. A randomization test with 999 bootstraps was applied to infer whether the observed correlation deviates from the null hypothesis of no correlation between geographic and genetic distance. Calculating geographic distance between BV and CA as straight line does not alter the results.

### Dispersal events

Dispersal rates between the study populations were estimated based on genetic as well as observational data. Given that the Buenaventura population was monitored intensively during 2008–2012 with a considerable proportion of individuals of known origin, we were able to estimate dispersal rates between our study sites reliably based on our observational data. This is particularly so because individuals are sedentary within an area of 100–120 ha [52]. Moreover, owing to our study on the reproductive behaviour of El Oro parakeets [36], we were able to estimate whether dispersal rates corresponded to actual gene flow rates. Rates of gene flow might be substantially lower than dispersal rates, particularly in a cooperative breeding system where only a fraction of birds actually breeds.

Dispersal rates based on genetic data were assessed with the software BayesAss ver. 3.0 [53]. This software estimates recent dispersal rates by applying a Bayesian approach and without assuming Hardy-Weinberg equilibrium within populations. To assess whether dispersal rates differ between the study sites, we calculated dispersal rates between all sample localities. Four independent runs were conducted with 10^7^ MCMC steps, a thinning interval of 10^4^ steps and a burn-in period of 10^6^ steps varying the random seed number in each run. The mixing parameter for allele frequencies was set to a = 0.2 to guarantee reasonable acceptance rates and good mixing of chains. Convergence of each chain was further checked with the software Tracer ver. 1.5 [54].

### Past population demography and bottleneck analysis

Increasingly high deforestation rates since the beginning of industrialisation in the mid 20^th^ century accompanied by the loss of potential breeding places and foraging trees led to a strong reduction of the global population of the El Oro parakeet [33]. Ever since the El Oro parakeet was described in the 1980s [37], its global population estimate has been continuously reduced paralleling the reduction of remaining habitat [33,55]. Given the inherent uncertainty of population estimates based on habitat cover, we applied two different methods to assess the extent of population size reductions in the El Oro parakeet. First, we conducted a heterozygosity excess test and a mode-shift test with the software BOTTLENECK ver.1.2.02 [56]. Both of these tests can detect recent bottleneck events in case the population has not yet re-attained mutation-drift equilibrium [57]. Because population structure may spuriously infer a bottleneck signal, heterozygosity excess was tested for several subsets of our data set with individuals assigned to their hatching place. Heterozygosity excess was tested separately for the genetic clusters inferred by the Structure analysis assigning individuals of BV_North_ and CA to one cluster (n = 142) and individuals of BV_South_ to a second cluster (n = 107), for the whole BV population (n = 233) and for the whole data set (n = 249) including individuals of CA. All three available mutation models were used: infinites alleles model (IAM), the single step mutation model (SMM) and the two-phase mutation model (TPM). For TPM, default values were used with 70% single step mutations and 30% multistep mutations with a variance of σ^2^ = 30. Probability of heterozygosity excess was estimated with the sign test and with the Wilcoxon test based on 10^5^ bootstrap iterations.

Second, we assessed potential past population decline with a Bayesian coalescent-based model implemented in MSVAR 1.3 [58]. The model infers population size changes by quantifying current N_0_ and ancestral N_1_ effective population size and the time T since the population size started changing. Probability estimates of parameters are assessed through MCMC simulations based on allele frequencies assuming a SMM model with mutation rate θ = 2N_0_μ where μ is the per locus mutation rate. Three models were run with wide priors set to a log-normal scale assuming different scenarios of population size change (S1 Table) to ensure that prior information does not bias model estimates towards a population bottleneck scenario. To keep time expenditure of calculations as small as possible without losing too much information, we used a data set comprising only those individuals of BV that were recruited to the population (n = 148), i.e. individuals that were captured as adults, or nestlings that were re-captured at least once, and the 13 adults of CA. We assumed a generation time of six years because individuals start breeding when two to three years old at the earliest and more than half of the known breeders keep their breeding position for three or more years (unpubl. data). Each chain was run for 4.5 ∙ 10^8^ iterations sampling from the chain every 1.5 ∙ 10^4^ steps yielding an output of 3 ∙ 10^4^ iterations for each run. The first 5 ∙ 10^3^ iterations were discarded from each run. Convergence of chains was tested with the Gelman-Rubin statistic as implemented in the R package BOA [59]. The median and the mode plus 90% highest probability density (HPD) intervals of all parameters were calculated with the R package *locfit* [60]. R code was partly taken from [61].

## Results

### Genetic diversity

Genetic diversity measured as observed heterozygosity was relatively high in all three study sites (Table 1). We found no evidence for non-random mating as F_IS_ indices were all near zero. Furthermore, genetic diversity in terms of allelic richness is similar for all three study sites.

**Table 1:** Genetic diversity calculated as mean (± SE) for each sampling location and for the whole data set (N = sample size).

Sampling location	N	H_e_	H_o_	AR	F_IS_
BV_North_	126	0.68 (± 0.02)	0.71 (± 0.03)	4.75 (± 0.33)	-0.049 (± 0.020)
BV_South_	107	0.63 (± 0.02)	0.64 (± 0.03)	4.42 (± 0.34)	-0.005 (± 0.020)
CA	16	0.61 (± 0.04)	0.68 (± 0.04)	4.36 (± 0.28)	-0.105 (± 0.031)
**Total**	**249**	**0.64 (± 0.02)**	**0.67 (± 0.02)**		**-0.053 (± 0.015)**

Estimates of genetic diversity include the expected (H_e_) and observed (H_o_) heterozygosity, allelic richness (AR) and the fixation index (F_IS_). BV_North_ = northern area of Buenaventura, BV_South_ = southern area of Buenaventura, CA = Cerro Azul.

### Population genetic structure

#### Genetic differentiation between local populations based on G_ST_ and D

Even though geographical distance between BV_South_ and BV_North_ is small (approximately 3 km), we found significant genetic differentiation between these two areas (Table 2). Likewise, we found small but significant genetic differentiation on a larger spatial scale between BV_North_ and CA as well as between BV_South_ and CA (Table 2). These results did not deviate qualitatively between the complete and reduced data set (Table 2, S2 Table). Yet, genetic differentiation was slightly lower when calculated for the reduced data set, particularly for estimates of genetic differentiation between BV_South_ and BV_North_ and between BV_North_ and CA (S2 Table).

**Table 2:** Pairwise genetic differentiation measured as Jost´s D and G_ST_ and bias-corrected for small sample sizes (est).

		D_est_	G_ST_est_
BV_South_	BV_North_	0.040 (0.033–0.059)	0.019 (0.017–0.027)
BV_South_	CA	0.059 (0.046–0.117)	0.035 (0.030–0.061)
BV_North_	CA	0.056 (0.044–0.122)	0.030 (0.025–0.055)

Confidence intervals inferred through bootstrapping are given in brackets. Values of genetic differentiation were measured for n = 249 between the northern area of Buenaventura (BV_North_), the southern area of Buenaventura (BV_South_) and Cerro Azul (CA).

#### Genetic cluster analyses

**Fig 2:**
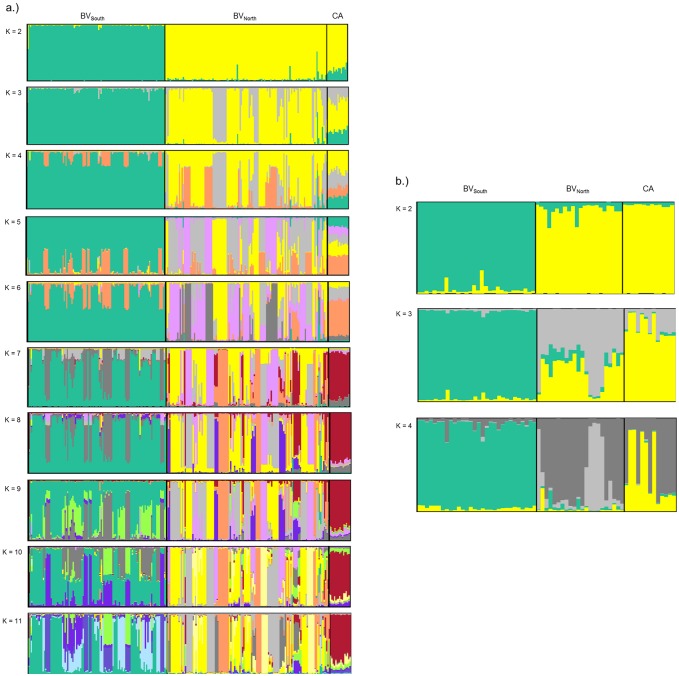
Genetic structure within the three study sites as inferred from the Structure analyses. Study sites comprise the southern area of Buenaventura (BV_South_), the northern area of Buenaventura (BV_North_) and Cerro Azul (CA). Each bar corresponds to an individual´s probability of belonging to a specific genetic cluster. Distinct clusters are assigned to different colours. Displayed are the results of Bayesian clustering analysis with prior information on sampling location for several numbers of K clusters. Presented are the results obtained a.) for the complete data set (n = 249) and b.) for the reduced data set (n = 65). The highest probability of clusters according to Evanno´s delta K was K = 2 for both data sets and K = 4 and K = 11 for the reduced and complete data set, respectively, using highest mean posterior likelihood.

The clustering analyses revealed clearly separated genetic clusters between BV_South_ and BV_North_ for both data sets (Fig 2a and 2b). In contrast, genetic sub-structuring between CA und BV_North_ was less clear as some individuals from both populations were assigned to the same genetic cluster (Fig 2). Moreover, the division between both localities was only evident for the complete data set at higher numbers of K (K ≥ 7) (Fig 2a). As the mean probability tends to overestimate the number of genetic clusters, we also present the maximum number of clusters as inferred by delta K (S1 Fig). Our results were independent of whether prior information about sampling location was given or not, we therefore only report results from runs with prior information. The high number of clusters found within the complete data set is most probably attributable to the breeding system of El Oro parakeets. High relatedness within kin clusters can result in an overestimation of K [62] and may explain the high number of clusters inferred using the complete data set. Nevertheless, our data reveals a clear separation between the northern and southern areas of Buenaventura and a slightly weaker separation between BV_North_ and CA.

The Geneland analysis based on the reduced data set revealed similar results as the Structure analysis with results based on the mean likelihood estimation. All ten runs showed a modal number of K = 4 genetic clusters for the reduced data set, which also corresponds to the second peak found using the Evanno´s deltaK method with Structure (S1b Fig). Individuals from BV_South_ and BV_North_ were assigned to separate genetic clusters, although two females known to have dispersed from BV_South_ to BV_North_ were correctly assigned to their population of origin (S2 Fig). In contrast to the Structure analysis, BV_North_ and CA showed a clear division with individuals from both sites assigned to separate genetic clusters. Note that results are only reported for the reduced data set because results of the analyses conducted with the complete data set showed the presence of ghost populations (i.e. no individuals assigned to some of the inferred genetic clusters). This pattern was found irrespective of model assumptions, for example correlated or uncorrelated allele frequency, and is probably due to the high relatedness and the spatially clustered structure within this data set.

#### Isolation by distance

Birds were genetically more differentiated the more distant their origins were. This is true for individuals within Buenaventura (IBD between BV_North_ and BV_South_: r = 0.19, p < 0.001, n = 233) as well as for individuals between BV and CA (r = 0.11, p = 0.003, n = 249). In both cases, the IBD results were similar to the correlation found for individuals from BV_South_ and CA (r = 0.19, p = 0.001, n = 124) indicating that even on these small spatial scale, geographic distance may hamper genetic exchange between individuals.

A Mantel test conducted for breeders of BV_South_ and BV_North_ revealed a similarly strong correlation as for the complete data set (r = 0.19, p < 0.001, n = 52) suggesting that the differentiation is not a result of the high relatedness within breeding groups, but may indeed be due to low dispersal rates and isolation by distance. We detected no correlation between geographic and genetic distance for breeders within subpopulations (BV_North_: r = -0.13, p = 0.08, n = 24; BV_South_: r = 0.11, p = 0.06; n = 28), showing that the variance in geographic distance within our sampling locations did not influence our results.

### Dispersal behaviour

Our observational data revealed that dispersal events between BV_North_ and BV_South_ are rare. During four years, we only detected five individuals crossing sites within the Buenaventura reserve: two females dispersed from BV_South_ to BV_North_ and two females and one male dispersed from BV_North_ to BV_South_. Taking only recruited individuals into account (n = 148), this corresponds to a dispersal rate of 3.4% in four years. Importantly, only the two females which dispersed to BV_North_ bred and produced one chick each. Hence, the proportion of dispersal events actually resulting in gene flow and admixture between BV_North_ and BV_South_ decreases to 1.4% (2 of 148 recruited individuals) during the study period of four years. These findings indicate existing but probably low rates of gene flow across a 3 km wide valley.

Similarly to our observational data, inference of dispersal rates from genetic data revealed low rates of dispersal between BV_North_ and BV_South_. Based on the complete data set, the proportion of migrants per generation was 0.02 for both dispersal directions within Buenaventura (Table 3). In contrast, a moderately high proportion of individuals dispersing from BV_North_ to CA were detected. Wide confidence intervals for dispersal rates from BV_South_ to CA and from CA to BV_North_ indicate that evaluation of dispersal rates were not possible in these cases. Importantly, the proportion of residents was very high for BV_South_ and considerably lower for BV_North_ and CA (Table 3). In summary, our observational data as well as estimations based on genetic data indicate that dispersal rates are low between the study sites, particularly so between BV_North_ and BV_South_.

**Table 3:** Dispersal rates between the sampling locations inferred by BayesAss.

**Sampling location**	**Sample size**	**Proportion non-migrants**	**95%-CI**
BV_South_	108	0.971	0.949–0.992
BV_North_	125	0.859	0.828–0.891
CA	16	0.689	0.649–0.730
**Source population**	**Target population**	**Proportion migrants**	**95%-CI**
BV_South_	BV_North_	0.019	0.001–0.036
BV_North_	BV_South_	0.020	0.002–0.038
BV_South_	CA	0.011	-0.002–0.024
CA	BV_South_	0.147	0.016–0.278
BV_North_	CA	0.121	0.091–0.150
CA	BV_North_	0.164	0.038–0.289

Given are the proportion of residents in each locality (upper table) and the proportion of migrants per generation between localities (lower table) for the complete data set of n = 249 individuals. Specified are the mean values and their confidence intervals averaged over four independent MCMC runs. BV_North_ = northern area of Buenaventura, BV_South_ = southern area of Buenaventura, CA = Cerro Azul.

### Population demography and bottleneck analysis

We detected significant heterozygosity excess within the subpopulations (Table 4). Evidence of population decline was consistently detected assuming the IAM or the TPM model (Table 4). In contrast, assuming a SMM model, we only found significant heterozygosity excess if pooling individuals captured in Buenaventura or if pooling all individuals (Table 4). We detected no deviation from an L-shaped distribution of allele frequencies for any sample used in the analyses. Nevertheless, as heterozygosity excess was detected irrespectively of the population structure assumed, our results provide strong evidence for the presence of a recent bottleneck within the El Oro parakeet population.

**Table 4:** Significance levels for Wilcoxon test and Sign test for a recent bottleneck event measured as heterozygosity excess and assuming different mutation models.

	Wilcoxon-Test			Sign-Test		
	IAM^1^	TPM^2^	SMM^3^	IAM^1^	TPM^2^	SMM^3^
BV_South_ (n = 107)	**< 0.001**	**< 0.001**	0.89	**< 0.001**	**0.006**	0.07
BV_North_ + CA (n = 142)	**< 0.001**	**< 0.001**	0.58	**< 0.001**	**0.006**	0.29
BV (n = 233)	**< 0.001**	**< 0.001**	0.95	**< 0.001**	**< 0.001**	**0.02**
BV + CA (n = 249)	**< 0.001**	**< 0.001**	0.96	**< 0.001**	**< 0.001**	**0.02**

^1^ IAM: Infinite alleles mutation model

^2^ TPM: Two-phase mutation model (with 70% single step mutations and variance of σ = 30)

^3^ SMM: Single step mutation model

The complete data set (n = 249) as well as different subsets of the data were tested (BV_North_ = northern area of Buenaventura, BV_South_ = southern area of Buenaventura, CA = Cerro Azul). Significance levels below 0.05 are given in bold. Significance levels reported for the Wilcoxon test are obtained from the one-tailed test for heterozygosity excess.

We obtained similar results of a population contraction using MSVAR simulations. Gelman-Rubin statistic indicated good convergence of all chains with point estimates of potential scale reduction factors < 1.1 [63]. Modal values and 90%-HPD intervals were N_0_ = 3.13 (1.12–4.04) for the current population size and N_1_ = 3.68 (3.19–4.21) for the ancestral population size. This corresponds to a median current population size of approximately 750 individuals and an ancestral median population size of approximately 5000 individuals, which indicates a 7-fold decrease in population size. Time since population size started decreasing was estimated to approximately 380 years with a mode of T = 2.72 (0.22–4.94). Median mutation rate was 3 ∙ 10^−4^ corresponding to a mode of θ = -3.53 (-3.94 - -3.12). Large confidence intervals particularly for parameter estimates of the timing of population decline but also for parameter estimates of the current population size indicate some uncertainty in the estimation of these parameters. Yet, the analyses give strong support for a decline of the El Oro parakeet population.

## Discussion

We detected low but significant genetic differentiation between all three study sites. Even on a small spatial scale as within the BV reserve genetic distance was increasing with geographic distance. Together with the observation of low dispersal rates between the study sites, our findings suggest that effective dispersal and gene flow rates are extremely low given the small geographic scale considered. Interestingly, dispersal rates as well as population structure inferred from individual genotypes revealed similar genetic sub-structuring between the two study sites within the Buenaventura reserve (~3 km distance) as between the northern area of Buenaventura and Cerro Azul (~13 km distance) indicating that geographic barriers may influence fine-scale genetic structure in the El Oro parakeet.

The genetic divergence and the existence of IBD between our subpopulations indicate that even small geographic distances limit dispersal and induce genetic differentiation between El Oro parakeet populations. This is surprising since parrots are usually thought to be highly mobile birds with often high levels of gene flow between distant populations [64,65]. Although low dispersal affinity might be expected in species with exceptionally small distribution ranges and high habitat specialization like in the El Oro parakeet, on comparable spatial scales, similar amounts of differentiation were found for strongly territorial understory birds, like the chestnut-backed antbird (*Myrmeciza exsul*) [19] and the Australian logrunner (*Orthonyx temminckii*) [18]. Thus the genetic differentiation found in our study seems to be exceptionally high given the small spatial scale considered. It also raises the question whether the observed genetic differentiation is due to geographic distance and low dispersal affinity alone.

In general, elevated levels of natal philopatry and low dispersal distances can be expected to induce fine-scale genetic sub-structuring in cooperatively breeding species [66–68]. It is thus likely that the complex social system of El Oro parakeets contributes to the genetic structure observed here. Low dispersal rates coupled with slow breeder turn-over entails that immigrants face long reproductive cues within flocks thereby reducing the rate of effective dispersal events. Accordingly, only two of five immigrants from either side of the valleys successfully bred and contributed to the genetic pool of the Buenaventura population. It seems thus reasonable that the complex social breeding system of El Oro parakeets with pro-longed philopatric phases lowers the chances of successful dispersal and induce genetic divergence between subpopulations.

The genetic population structure found between the study sites may be explained by drift effects caused by a recent bottleneck event [3,69,70]. Genetic differentiation due to habitat loss and random drift of alleles corresponds to the very strong (>95%) reduction of forest cover in western Ecuador [38]. Importantly, the excess of heterozygosity found within the study populations hints to a recent bottleneck event. Moreover, our coalescence-based bottleneck analysis indicates a population decline of approximately 90% supporting the assumption that the El Oro parakeet subpopulations have been exposed to strong drift effects. Divergence between subpopulations on the scale of 3–17 km may thus be explained by genetic drift following a population decline. Yet, it is noteworthy that genetic signals of a recent bottleneck event will be readily diminished by even low rates of effective dispersal [71]. It is thus feasible that the observed fine-scale genetic structure is a combined effect of low effective dispersal rates and drift due to the recent population decline.

Population structure inferred from individual genotypes as well as the indices of genetic differentiation revealed similar genetic sub-structuring between the two study sites within the Buenaventura reserve (~3 km distance) as between the northern area of Buenaventura and Cerro Azul (~13 km distance). Accordingly, we found low migration rates within the Buenaventura reserve. This finding is even more surprising since the valley separating the northern and southern parts of Buenaventura reserve is covered by forest seemingly providing good conditions for dispersal, whereas the region between the northern area of Buenaventura and Cerro Azul is covered by forest patches only. Despite this and although only three kilometre distance separates the northern and southern mountain ranges, our observational and genetic data indicate that El Oro parakeets rarely cross the valley. The genetic divergence found between individuals inhabiting opposite sides of the valley may be explicable by the biogeographic history of the Andean region. Dispersal barriers as river valleys and mountain ridges are considered to have played a central role in the allopatric speciation events during the Andean uplift and thereby have contributed to the high species diversity observed nowadays in this region [72]. Even narrow barriers preserve the genetic and ecological divergence between species in the Andes [22,26,29,30], although they were probably covered by suitable habitat in the past [29]. Although historical genetic samples are not available for the El Oro parakeet, past population monitoring showed that El Oro parakeets occupied the low-elevation forest from 600–800 m in Buenaventura three decades ago [37]. Given these observations, the biogeographic history of this region unlikely explains the relatively high genetic divergence and the low dispersal rates across the valley. Given that the valley was inhabited by El Oro parakeets in the past, the question arises why parrots now avoid low-elevation areas that were suitable in the past?

Ongoing population decline over the past decades, as shown by our bottleneck analyses, could have led to the withdrawal of El Oro parakeets from marginal habitat in the lower parts of the valley. The reduction of population size can lead to a re-assortment of the breeding population followed by the abandonment of marginal habitat [73]. The Buenaventura valley may represent such marginal habitat to El Oro parakeets because climatic conditions are slightly different than in the upper parts of the region, i.e. higher temperatures and lower humidity. Although a marginal habitat effect might be a plausible explanation for the abandonment of low-elevation habitat during the past decades, this conjecture does not explain why El Oro parakeets were not observed >1100m in Buenaventura in the 1980s, but can now be regularly observed there.

Although climatic data are scarce for this region, altered conditions due to climate change are known to shift cloud cover, and hence humidity, to higher elevations in tropical montane cloud forests [74–76] leading to elevational range shifts of bird communities [77,78]. Species of the genus Pyrrhura are considered highly sensitive to landscape structure and environmental gradients [34]. Increasingly dry conditions in the Buenaventura valley are reported by locals. Thus, climatic changes may have resulted in a continued upslope movement of El Oro parakeets ever since the species’ discovery in the 1980s [35,37,52], making previously suitable low-elevation areas a natural barrier to dispersal to this species in the recent past. Independent of whether humidity is the determining factor, the upslope range shift of El Oro parakeets fits the prediction that such shifts will be an inevitable consequence of proceeding climate change [79,80]. It is likely that such upslope movements have important long-term consequences for biodiversity as they will reduce gene flow among increasingly isolated populations in the Andes and montane tropics in general. Our data on El Oro parakeets indicate that increasing genetic differentiation will not only be evident in organism with low dispersal capacity [81] but also in mobile species like birds that occupy narrow elevational ranges.

### Current status and conservation implications

High deforestation rates are thought to pose an imminent threat especially to the survival of endemic species like the El Oro parakeet. Consensus estimates suggested a population decline of El Oro parakeets from approximately 8000–10000 individuals to approximately 1000 individuals during the last three decades [33,55]. Although our results do not allow making precise statements about current population sizes, they nevertheless support the assumption that the El Oro parakeet population has undergone a severe bottleneck. Moreover, we found evidence of a recent bottleneck, thereby supporting the assumption that accelerated deforestation rates during the last century induced the population decline of this species.

Population decline is often accompanied by adverse genetic effects like reduced genetic connectivity between habitat fragments and reduced genetic diversity [2]. In the El Oro parakeet, levels of heterozygosity were similar for all three subpopulations. Unfortunately, making inference on whether habitat disturbance and population decline has impacted genetic diversity is difficult because there are no historic references available [82]. However, on the species level, our estimates of genetic diversity are presumably optimistic as we have sampled the two largest known populations of El Oro parakeets.

El Oro parakeets strongly differ in their dispersal behaviour from other sympatric parrot species (like the bronze-winged parrot (*Pionus chalcopterus*) or the red-masked parakeet (*Aratinga erythrogenys*)) in that they are usually flying through the canopy, which probably makes them highly susceptible to habitat loss and fragmentation. The population structure analysis combined with spatial information revealed that individuals from CA and BV_North_, where forest cover is widely missing, build two separate genetic clusters. In contrast, the dispersal rates inferred from genetic data suggest that dispersal between Buenaventura and Cerro Azul is not completely disrupted. In accordance, the Structure analyses showed that individuals from Cerro Azul do not form a completely separated genetic cluster. Instead, a few individuals from the northern area of Buenaventura were assigned to the genetic cluster of Cerro Azul. These findings indicate that, even though forest cover is largely reduced in this area, dispersal is still ongoing. This may be explained by the observation that El Oro parakeets regularly cross pastures, usually by flying close to the ground and often by using single tree groups as stopover. Moreover, El Oro parakeets presumably residing in forest fragments between Cerro Azul and Buenaventura may facilitate the maintenance of gene flow between these areas.

Our findings highlight the importance to consider species behaviour and its dependence on ecological conditions when planning conservation actions. For El Oro parakeets, dispersal corridors through low-elevation areas would most probably not be very effective even if geographical distances were small. In contrast, re-building or maintaining small but regularly occurring habitat islands at the appropriate elevation may be a more effective way to maintain gene-flow between populations. Importantly, assuming that the upslope movement of El Oro parakeets, and probably other cloud forest specialists, continues in the future, securing gene flow will necessitate dispersal corridors at the upper limits of the species elevational range.

## Supporting Information

S1 Fig Mean log-likelihood LnP(K) (filled circles) and mean delta K (open circles) based on 15 replicates for each number of K clusters. (DOCX)

S2 Fig Genetic structure within the three study sites as inferred from the Geneland analysis. (DOCX)

S1 Table Prior and hyperprior parameters for runs conducted with MSVAR 1.3. (DOCX)

S2 Table Pairwise genetic differentiation measured as Jost´s D and G_ST_ for n = 65 individuals. (DOCX)

S3 Table Genotype table. Given are genotypes of each individual and sample location.
(XLSX)
