# Supplementary material for: Limited Dispersal and Significant Fine - Scale Genetic Structure in a Tropical Montane Parrot Species
Source: PLoS One. 2016 Dec 29;11(12):e0169165. doi: 10.1371/journal.pone.0169165 (PMC5199109; doi:10.1371/journal.pone.0169165)
Supplement: S1 Fig — (DOCX) [file pone.0169165.s001.docx]

**Figure S1a**

**Figure S1b**

**Figure S1** Mean log-likelihood LnP(K) (filled circles) and mean delta K (open circles) based on 15 replicates for each number of K clusters. Presented are results of clustering analyses with prior information about sampling location conducted with a.) the complete data set of n = 249 individuals and with b.) the reduced data set of n = 65 individuals. Error bars indicate standard deviations.
