# Supplementary material for: Limited Dispersal and Significant Fine - Scale Genetic Structure in a Tropical Montane Parrot Species
Source: PLoS One. 2016 Dec 29;11(12):e0169165. doi: 10.1371/journal.pone.0169165 (PMC5199109; doi:10.1371/journal.pone.0169165)
Supplement: S2 Fig — (DOCX) [file pone.0169165.s002.docx]

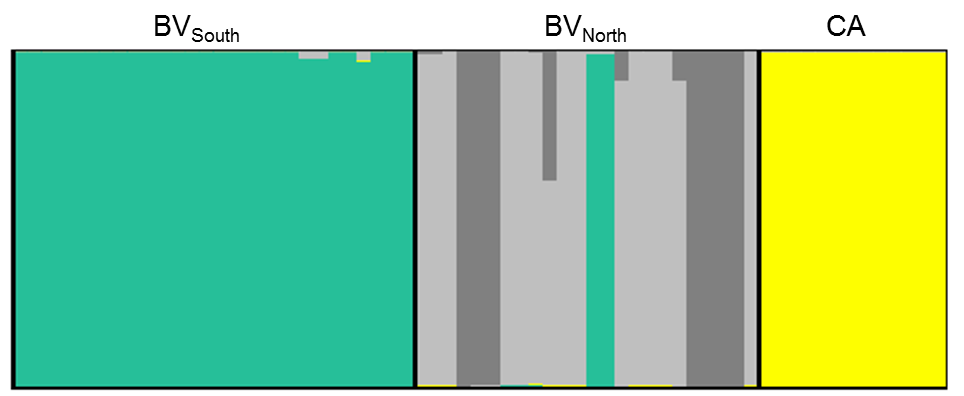


**Figure S2** **Genetic structure within the three study sites as inferred from the Geneland analysis**. Study sites comprise the southern area of Buenaventura (BV_South_)_,_ the northern area of Buenaventura (BV_North_) and Cerro Azul (CA). Each bar corresponds to an individual´s probability of belonging to a specific genetic cluster. Each of the four genetic clusters is presented in a different colour. Displayed are the results of Bayesian clustering analysis for the reduced data set (n = 65).
