# Supplementary material for: Limited Dispersal and Significant Fine - Scale Genetic Structure in a Tropical Montane Parrot Species
Source: PLoS One. 2016 Dec 29;11(12):e0169165. doi: 10.1371/journal.pone.0169165 (PMC5199109; doi:10.1371/journal.pone.0169165)
Supplement: S1 Table — (DOCX) [file pone.0169165.s003.docx]

**Table S1:** Prior and hyperprior parameters for runs conducted with MSVAR 1.3. Specified are starting values for the mean and variance of the prior distributions and the means and variances (and their means and variances) of the hyperprior distributions for the following parameters: current effective population size N0, ancestral effective population size N1, mutation rate (Ѳ), and time since population size changed T. All values are in a log_10_ scale.

|  | **Starting values for prior** | | | | **Hyperpriors** | | | |
| --- | --- | --- | --- | --- | --- | --- | --- | --- |
|  | log(N0) | log(N1) | logӨ | log(T) | log(N1) | log(N0) | logӨ | log(T) |
| Run1 | 4 1 | 5 1 | -3.5 1 | 3 2 | 4 3 0 0.5 | 4 2 0 0.5 | -3.5 0.25 0 0.5 | 3 2 0 0.5 |
| Run2 | 4 1 | 5 1 | -3.5 1 | 2 1 | 4 2 0 0.5 | 4 2 0 0.5 | -3.5 0.25 0 0.5 | 3 2 0 0.5 |
| Run3 | 4 2 | 4 2 | -3.5 1 | 2 1 | 4 2 0 0.5 | 5 2 0 0.5 | -3.5 0.25 0 0.5 | 2 2 0 0.5 |
