# Supplementary material for: Limited Dispersal and Significant Fine - Scale Genetic Structure in a Tropical Montane Parrot Species
Source: PLoS One. 2016 Dec 29;11(12):e0169165. doi: 10.1371/journal.pone.0169165 (PMC5199109; doi:10.1371/journal.pone.0169165)
Supplement: S2 Table — (DOCX) [file pone.0169165.s004.docx]

**Table S2** Pairwise genetic differentiation measured as Jost´s D and G_ST_ and bias-corrected for small sample sizes (est). Confidence intervals inferred through bootstrapping are given in brackets. Values of genetic differentiation were measured for n = 65 between the northern area of Buenaventura (BV_North_), the southern area of Buenaventura (BV_South_) and Cerro Azul (CA).

|  |  | **D_est_** | **G_ST_est_** |
| --- | --- | --- | --- |
| BV_South_ | BV_North_ | 0.027 (0.024 - 0.088) | 0.017 (0.016 - 0.041) |
| BV_South_ | CA | 0.051 (0.042 - 0.123) | 0.030 (0.028 - 0.064) |
| BV_North_ | CA | 0.037 (0.038 - 0.129) | 0.025 (0.024 - 0.060) |
